# Deep learning super-resolution magnetic resonance spectroscopic imaging of brain metabolism and mutant isocitrate dehydrogenase glioma

**DOI:** 10.1093/noajnl/vdac071

**Published:** 2022-05-24

**Authors:** Xianqi Li, Bernhard Strasser, Ulf Neuberger, Philipp Vollmuth, Martin Bendszus, Wolfgang Wick, Jorg Dietrich, Tracy T Batchelor, Daniel P Cahill, Ovidiu C Andronesi

**Affiliations:** A. A. Martinos Center for Biomedical Imaging, Department of Radiology, Massachusetts General Hospital, Harvard Medical School, Boston, Massachusetts, USA; Department of Mathematical Sciences, Florida Institute of Technology, Melbourne, Florida, USA; A. A. Martinos Center for Biomedical Imaging, Department of Radiology, Massachusetts General Hospital, Harvard Medical School, Boston, Massachusetts, USA; Department of Neuroradiology, Heidelberg University Hospital, Heidelberg, Germany; Department of Neuroradiology, Heidelberg University Hospital, Heidelberg, Germany; Department of Neuroradiology, Heidelberg University Hospital, Heidelberg, Germany; Department of Neurology, Heidelberg University Hospital, Heidelberg, Germany; Department of Neurology, Massachusetts General Hospital, Harvard Medical School, Boston, Massachusetts, USA; Department of Neurology, Brigham and Women Hospital, Harvard Medical School, Boston, Massachusetts, USA; Dana-Farber Cancer Institute, Boston, Massachusetts, USA; Department of Neurosurgery, Massachusetts General Hospital, Harvard Medical School, Boston, Massachusetts, USA; A. A. Martinos Center for Biomedical Imaging, Department of Radiology, Massachusetts General Hospital, Harvard Medical School, Boston, Massachusetts, USA

**Keywords:** D-2-hydroxyglutarate, deep learning, glioma, isocitrate dehydrogenase, magnetic resonance spectroscopic imaging, super-resolution

## Abstract

**Background:**

Magnetic resonance spectroscopic imaging (MRSI) can be used in glioma patients to map the metabolic alterations associated with *IDH1,2* mutations that are central criteria for glioma diagnosis. The aim of this study was to achieve super-resolution (SR) MRSI using deep learning to image tumor metabolism in patients with mutant IDH glioma.

**Methods:**

We developed a deep learning method based on generative adversarial network (GAN) using Unet as generator network to upsample MRSI by a factor of 4. Neural networks were trained on simulated metabolic images from 75 glioma patients. The performance of deep neuronal networks was evaluated on MRSI data measured in 20 glioma patients and 10 healthy controls at 3T with a whole-brain 3D MRSI protocol optimized for detection of d-2-hydroxyglutarate (2HG). To further enhance structural details of metabolic maps we used prior information from high-resolution anatomical MR imaging. SR MRSI was compared to ground truth by Mann–Whitney *U*-test of peak signal-to-noise ratio (PSNR), structure similarity index measure (SSIM), feature-based similarity index measure (FSIM), and mean opinion score (MOS).

**Results:**

Deep learning SR improved PSNR by 17%, SSIM by 5%, FSIM by 7%, and MOS by 30% compared to conventional interpolation methods. In mutant IDH glioma patients proposed method provided the highest resolution for 2HG maps to clearly delineate tumor margins and tumor heterogeneity.

**Conclusions:**

Our results indicate that proposed deep learning methods are effective in enhancing spatial resolution of metabolite maps. Patient results suggest that this may have great clinical potential for image guided precision oncology therapy.

**Key Points**

- Develop a deep learning method for super-resolution MRSI using GAN deep neural networks to upsample metabolic maps by a factor of 4.
- Combine deep learning and variational methods to further improve the final super-resolution MRSI results.
- Demonstrate deep learning super-resolution MRSI in mutant IDH glioma patients to obtain the highest resolution of 2HG maps that delineate tumor margins and tumor heterogeneity.

**Importance of the Study**

Magnetic resonance spectroscopic imaging (MRSI) can be used in glioma patients to map the metabolic alterations associated with *IDH1,2* mutations that are central criteria for glioma diagnosis. The spatial and temporal patterns of MRSI metabolic lesions correlate with tumor progression, treatment response, and clinical outcome. Importantly, tumor appearance and dynamics in MRSI are different compared to anatomical MRI. However, because of low metabolite concentrations MRSI is acquired at low resolution (ie, larger voxels), which may result in reduced structural details, with less ability to probe tumor heterogeneity, small and early tumors. To address this challenge, in this work we developed and investigated the performance of deep learning methods for super-resolution MRSI in glioma patients. Such methods can be used for image guided precision oncology therapy.

Magnetic resonance spectroscopic imaging (MRSI) can be used in glioma patients to map the metabolic alterations associated with *IDH1,2* mutations that are central criteria for glioma diagnosis in the most recent classification 2021 WHO of brain tumors.^1^ The hallmark metabolic alteration of cancer *IDH1,2* mutations is the de novo overproduction of d-2-hydroxyglutarate (2HG)^2^ as an “oncometabolite” that drives the epigenome^3^ and microenvironment^4^ toward tumor progression. Due to its high specificity, 2HG is a valuable imaging biomarker for diagnosing,^5,6^ monitoring, assessing tumor burden,^7^ treatment response,^8,9^ and pharmacodynamics of targeted therapy in mutant IDH1 glioma.^10^

^1^H-MRSI in the human brain can measure up to 20 metabolites^11^ that probe-specific molecular mechanisms in neurological diseases, including brain tumors.^12^ The spatial and temporal patterns of MRSI metabolic lesions correlate with tumor progression, treatment response, and clinical outcome.^9,13,14^ Importantly, tumor appearance and dynamics in MRSI are different compared to anatomical MRI.^15^ However, because of low metabolite concentrations MRSI is acquired at low resolution (ie, larger voxels) to compensate for lower SNR. In this scenario, structural detail is reduced and small lesions might be missed since boundaries of lesions are blurred. An efficient approach for improving spatial resolution is to upsample MRSI using super-resolution (SR) methods^16^ that recover fine structural details. Recently, SR MRSI has been demonstrated using patch-based,^17^ feature nonlocal means (FNLM),^18^ and deep learning.^19,20^

In this work, we developed and investigated the performance of deep learning methods for SR MRSI, particularly in glioma patients. The earlier work of Iqbal et al.^19^ in healthy volunteers showed upsampled MRSI by deep learning in combination with prior anatomical MRI. Here, inspired by the work of Iqbal et al., we extended the Unet model^21^ with the framework of the generative adversarial network (GAN)^22^ in order to build more robustness for larger variability in patient data.

In our patient study, we took a 2-step approach to upsample low-resolution (LS) MRSI: (1) using deep learning alone (DLmethod_1), and (2) combining deep learning with prior high-resolution (HR) MRI. As metabolic and anatomical lesions can differ in shape, contrast, and texture, this raises the possibility that spurious spatial features can be introduced from anatomical MRI into MRSI. Our 2-step approach has been motivated by the need to deal with this circumstance. In addition, we studied 2 ways of combining deep learning with prior HR MRI: (1) using a sequential method (DLmethod_2) in which MRSI is first upsampled by deep learning and subsequently reinterpolated by FNLM weights based on prior MRI, and (2) using a simultaneous method (DLmethod_3) in which both initialized SR MRSI and HR MRI data are input together in the deep learning model. For the first method, we leveraged the FNLM, which we showed to be robust in glioma patients with respect of introducing false structural details from MRI into MRSI.^18^ We hypothesize that deep learning may provide better initialization for upsampling SR methods compared to conventional interpolation methods (bicubic, spline) or more advanced variational methods such as weighted total variation (wTV).

A reliable SR MRSI method may increase throughput and feasibility of metabolic imaging in clinical setup. The availability of MR scanners is limited and minimizing the acquisition time is key in providing advanced imaging to more patients and decrease the costs. On the other hand, computing power has advanced greatly, it is more ubiquitous and has lower costs, hence allowing to efficiently shift the burden from image acquisition to image processing. Here, we aim to capitalize on the increased compute performance with the goal to improve the quality and time efficiency for SR MRSI metabolic imaging for neuro-oncology applications in glioma patients.

## Materials and Methods

### Training Data Simulation

One of the obstacles to employ deep neural network (DNN) to upsample metabolic MRSI maps is that there are not sufficient HR training data measured in patients. The need of training data becomes even more demanding when more convolutional layers are used in DNN. Measurement of HR MRSI is prohibitive in patients due to long measurement times and lack of wide availability of pulse sequences that can acquire HR MRSI efficiently.^23^ To solve this issue, we resorted to simulations of realistic MRSI metabolic maps in order to generate sufficient training datasets similar to our prior work.^18^ To obtain patient training MRSI datasets we started from anatomical FLAIR and MEMPRAGE images acquired with 1 mm isotropic resolution in 75 glioma patients. FLAIR images were used to segment tumors (TM) by ITK_SNAP.^24^ The healthy part of brain was segmented by FSL^25^ on MPRAGE images into white matter (WM), gray matter (GM), and corticospinal fluid (CSF). The tumor and healthy brain segmentations were combined to generate very high-resolution (VHR) MRSI maps according to


$$\begin{array}{l} MRSI_{VHR}=0.1\times GM+0.12\times WM+0\times CSF+\tau \times TM \end{array}$$

where *τ* is chosen from [0.2, 0.3, 0.4, 0.5, 0.6, 0.7, 0.8], and the overall image intensity range is normalized to 1. The VHR MRSI data of size (256 × 256 × 176) were down sampled subsequently in *k*-space to generate LR MRSI data (46 × 46 × 32). The ground truth HR MRSI data (184 × 184 × 128), corresponding to LR–HR upsampling factor of 4, were generated in 2 ways: (1) by down sampling MRSI_VHR_ in *k*-space, and (2) by upsampling the LR MRSI with the FNLM SR pipeline that has been demonstrated in ^18^. The first type of HR MRSI data has sharper structural details, similar to anatomical imaging, and have been used to train the neural networks for the DLmethod_2 and DLmethod_3. The second type of HR MRSI has less sharp anatomical edges, similar to the quality of MRSI acquired with advanced HR protocols,^26–29^ and have been used to train the neural networks for DLmethod_1. In total each 9600 datasets for the 2 types of ground truth HR were simulated for training and validation. In addition, we employed data augmentation that flips and randomly crops the minibatch data during training process.

### Theory

Given a preprocessed LR metabolite map ***I***^LR^, our goal is to find an operator *F* such that *F*(***I***^LR^) is similar enough to the ground truth metabolite map ***I***^HR^, where *F* could be linear or nonlinear. Mathematically, this can be written as


$$I^{\text{LR}}=F^{-1}(I^{\text{HR}})+n$$ (1)

where *F*^−1^ represents an inverse operator of *F* and **n** is the noise term. For the deep learning method, *F* is usually a nonlinear operator. For the variational model-based reconstruction method, it is usually a linear operator.

#### Unet and GAN architectures.—

To upsample ***I***^LR^, we explore 2 types of neural networks, where the first is the so-called Unet typically used for image segmentation. Considering that Unet performs pixel-wise transformations, it can be reframed for SR tasks as initially recognized by Iqbal et al.^19^ The dense Unet architecture implemented by Iqbal et al. is able to carry over features from layer to layer and allow feature reuse throughout the network.^30^ However, the extensive feature reuse could introduce spurious features more easily considering the fact the anatomical images are also given as the input to the neural network, in particular when there are differences between metabolic and anatomical images in patients, such as different texture or extent of lesions. In our implementation instead of introducing densely connected networks in the Unet architecture we employed a similar neural network architecture as Ronneberger et al.,^21^ where the Unet architecture consists of 2 paths. The contraction path, which aims at extracting significant features from the input images, is mainly involved with standard convolutional and max pooling layers. The expanding path works toward identifying important features locally from a finer resolution using transposed convolutions and feature concatenation. Since no dense layer is involved and only convolutional layers are used, our Unet is essentially a fully convolutional network. The input for Unet is an initialized SR ***I***^ISR^ by bicubic interpolation. The activation function, Relu, is employed in all layers.

Furthermore, to augment the performance of the Unet we introduced a discriminator network,^31^ which formulates a GAN by treating the Unet as the generator network. The generative adversarial model typically involves 2 components, the generator network ***G***_*θ*_ and the discriminator network ***D***_*η*_, where *θ* denotes the parameters in generator network and *η* denotes the parameters in discriminator network. The parameters are comprised by weights (*W*) and biases (*B*) in all the layers (*L*) of the network that are learned during training. Our ultimate goal is to learn a generator function ***G*** that is able to reconstruct from the initialized SR image (***I***^ISR^) an image ***G***(***I***^ISR^) such that the reconstructed image is as similar as possible to the ground truth metabolite map ***I***^HR^. However, for GAN the generator network ***G***_*θ*_ is trained to generate SR metabolite maps so that the simultaneously trained differential discriminator ***D***_*η*_ cannot distinguish the generated maps from the ground truth HR metabolite maps, which can be solved via the following saddle point optimization problem


$$\begin{array}{l} \underset{\theta }{min}\;\;\underset{\eta }{max}\;\left\{E_{\;I^{HR}∼P_{train}(I^{HR})}\left[log\;D_{\eta }(I^{HR})\right]+E_{\;I^{LR}∼P_{G}(I^{LR})}\left[log\;\;\;\left(1-D_{\eta }\left(G_{\theta }(I^{LR})\right)\right)\right]\right\} \end{array}$$ (2)

**Figure 1.**
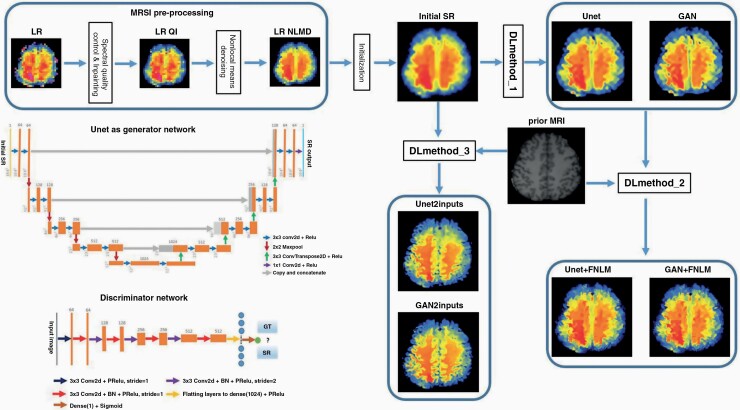
Flowchart diagram of deep learning super-resolution (SR) for magnetic resonance spectroscopic imaging (MRSI). There are 3 main blocks: (1) the low-resolution metabolic maps are first filtered using spectral quality criteria, inpainted and denoised, (2) the denoised maps are interpolated to produce the initial SR maps, and (3) the initialized SR is input in the deep neural networks (Unet or GAN) to obtain the final SR image. The last block can be run in 3 ways: (1) for DLmethod_1 only the initial MRSI and deep neural networks are used, (2) for DLmethod_2 the results of DLmethod_1 are further improved by subsequent feature nonlocal means (FNLM) with prior MRI, and (3) for DLmethod_3 the initial MRSI and prior MRI are both input in the deep neural networks. The architecture of the generator network (Unet) and discriminator network that are part of GAN are shown on the bottom left.

The architecture of the proposed Unet–GAN model is shown in Figure 1, which basically follows architectural guidelines in ^31^. Throughout the generator network, ParametricReLU (PRelu) activation is utilized but no max pooling is involved. In total, there are 8 convolutional layers in the discriminator network, in which 3 × 3 convolutional kernels are used. The number of kernels is increased from 64 to 512 kernels. Image size is reduced by strided convolutions when the number of features is doubled. To obtain a probability for sample classification, 2 dense layers and a final sigmoid activation function are employed following the final obtained feature maps.

The cost function for the optimization problem is defined as


$$\begin{array}{l} \frac{1}{N}\sum_{n=1}^{N}\left(\sum \sum {\left(I^{HR}-G_{\theta }(I^{LR})\right)}^{2}+\lambda \;log\left(-D_{\eta }\left(G_{\theta }(\;I^{LR})\right)\right)\right) \end{array}$$ (3)

which consists of 2 components, mean square error (MSE) and adversarial loss over *N* training samples, where *λ* ≥ 0 is the regularization parameter. When only Unet (the generator network) is used, we set *λ* = 0, that is, only MSE is used in the cost function for computing pixel-wise loss. The cost function is referred to as the perceptual loss function when *λ* > 0, which contributes significantly to the superior performance by GAN. Both Unet and GAN networks were trained in 2 ways: (1) using as input only the MRSI data (DLmethod_1 and DLmethod_2), and (2) using as input both the MRSI and prior MRI (DLmethod_3). Note that DLmethod_2 is DLmethod_1 combined with FNLM, hence for both methods the training is done similarly. The block diagram for training of all deep learning methods is shown in Figure 1.

We trained our networks on a PowerEdge R730 server (Dell) with 24 CPU cores (Intel Xeon E5-2687W v4 3.0 GHz) and 128 GB RAM (RDIMM, 2400 MT/s) running Linux Centos 7.6 using Tensorflow packages in Python 3.6. Unet was trained with batch size of 16 until convergence reached, which took 20k iterations. The Adam optimizer^32^ was used with a learning rate of 1e−4 for minimizing the cost function. GAN employed Unet as a pretrained model and was trained with batch size of 16 and the convergence reached after 20k iterations too. The Adam optimizer was used with a learning rate of 1e−5 for first 10k iterations and 1e−6 for last 10k iterations, where the first and second momentum terms were set 0.9 and 0.999, respectively.

### Measured MRSI Data Acquisition and Processing

Twenty mutant *IDH1* glioma patients and 10 healthy control subjects were recruited for this study. All participants gave written informed consent with an IRB approved protocol. *IDH1*-mutational status in patients was tested by anti-human R132H antibody (DIANOVA).^33^

Whole-brain 3D MR spectroscopic imaging data were acquired at 3T on a Tim Trio scanner (Siemens Medical Solutions) equipped with a 32-channel head coil and a gradient coil capable of 40 mT m maximum amplitude (200 mT m s slew rate), using a pulse sequence optimized for 2HG detection with adiabatic spin echo excitation and weighted 3D stack-of-spiral *k*-space encoding. Acquisition, reconstruction and processing were performed as described in Supplementary Material and further detailed in ref. ^18^: (1) metabolic maps from 20 brain metabolites were obtained in all subjects, and (2) 2 metabolic ratios that have high contrast to noise ratio for tumors were calculated in patients: HGG = ([2HG] + [Glutamine])/[Glutamate] and TCN = [total Choline]/[total *N*-acetyl-aspartate].

### Image Quality Metrics and Statistical Analysis

Results of the deep learning SR methods were compared to the ground truth using image quality metrics^34^: peak signal-to-noise ratio (PSNR), structural similarity index measure (SSIM), feature similarity index measure (FSIM), and mean opinion score (MOS). For MOS 3 experts were asked to evaluate and compare the SR images relative to ground truth based on 4 criteria scored from 1 (worst) to 5 (best) as following: (1) tumor boundaries, (2) local texture and intensity distribution inside the tumor, (3) healthy brain anatomical landmarks such as ventricles, sulci, gray–white matter border, and (4) healthy brain local texture and intensity distribution. The Mann–Whitney *U*-test in Matlab (Mathworks) was used to verify for statistically significant differences between the image quality metrics of different methods.

## Results

**Figure 2.**
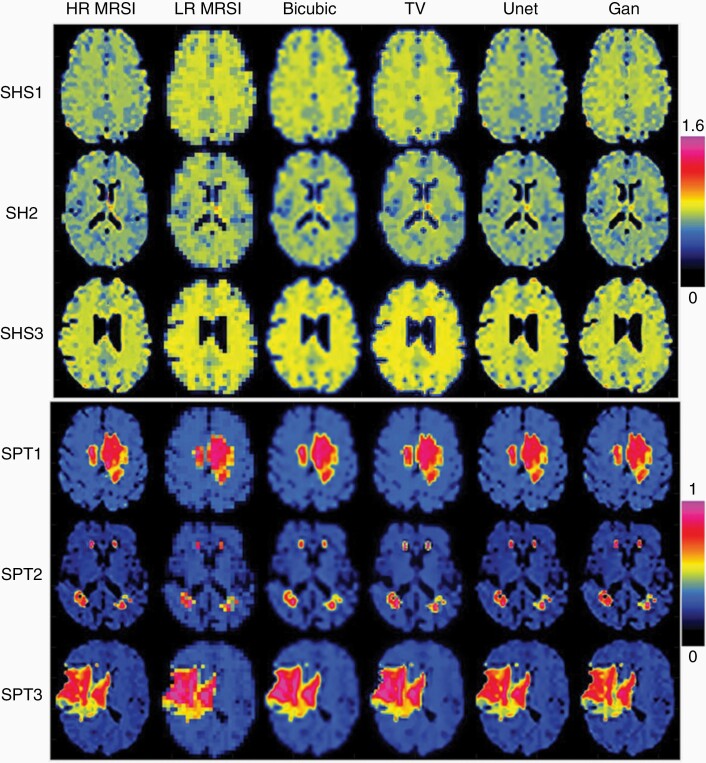
Super-resolution magnetic resonance spectroscopic imaging (MRSI) without prior MRI (DLmethod_1) in simulated NAA maps in healthy subjects (SHS) and simulated d-2-hydroxyglutarate (2HG) maps in patients (SPT) with glioma. Results obtained by Unet and GAN are compared to conventional interpolation methods (bicubic and total variation). Examples from 3 simulated healthy subjects and 3 simulated patients are shown, from left to right: high-resolution (HR) ground truth MRSI (184 × 184), low-resolution (LR) MRSI (46 × 46), upsampled MRSI (184 × 184) obtained by bicubic, total variation (TV), Unet, and GAN.

First, we investigated the performance of MRSI upsampling using Unet and GAN without prior MRI (DLmethod_1). Results obtained in 3 representative healthy subjects and 3 patients are shown in Figure 2. It can be seen that both Unet and GAN outperform conventional non-AI methods (bicubic and total variation), providing SR maps that have similar structural details as the ground truth. In particular, SR maps obtained by GAN have sharper edges for brain anatomical structure such as the gray–white matter boundary, compared to Unet that shows some over-smoothing of this boundary. Figure 2 also presents results from 3 representative patients, where Unet and GAN provide sharper tumor edges and richer texture inside the tumors compared to conventional methods, but more similar to the ground truth. In Supplementary Figure 1, the zoomed tumor region in the same 3 patients shows that GAN conforms slightly better than Unet with the ground truth tumor boundary. The evaluation of image quality metrics is summarized in Table 1 and Supplementary Figure 2. Deep learning scored higher than conventional interpolation, increasing PSNR by 17%, SSIM by 5%, FSIM by 7%, and MOS by 30%. The quantitative scores (PSNR, SSIM, and FSIM) are slightly higher for Unet than GAN, while the qualitative MOS score is higher for GAN than Unet.

**Table 1.** Performance of the deep learning methods alone (DLmethod_1) and conventional interpolation for upsampling MRSI

Method→	Conventional		DLmethod_1	
IQM↓	Bicubic	TV	UNet	GAN
PSNR	28.57	28.25	33.10*	30.83*
SSIM	0.921	0.868	0.964*	0.934*
FSIM	0.901	0.892	0.964*	0.939*
MOS	3.42	3.35	4.31*	4.42*

Image quality metrics (IQM) of super-resolution MRSI were calculated relative to the simulated ground truth high-resolution MRSI. The largest values are shown in bold. Values that are statistically significant for deep learning compared to conventional methods are indicated by asterisk. Boxplots of IQM are shown in Supplementary Figure 2. FSIM, feature similarity index measure; MOS, mean opinion score; MRSI, magnetic resonance spectroscopic imaging; PSNR, peak signal-to-noise ratio; SSIM, structure similarity index measure.

**Figure 3.**
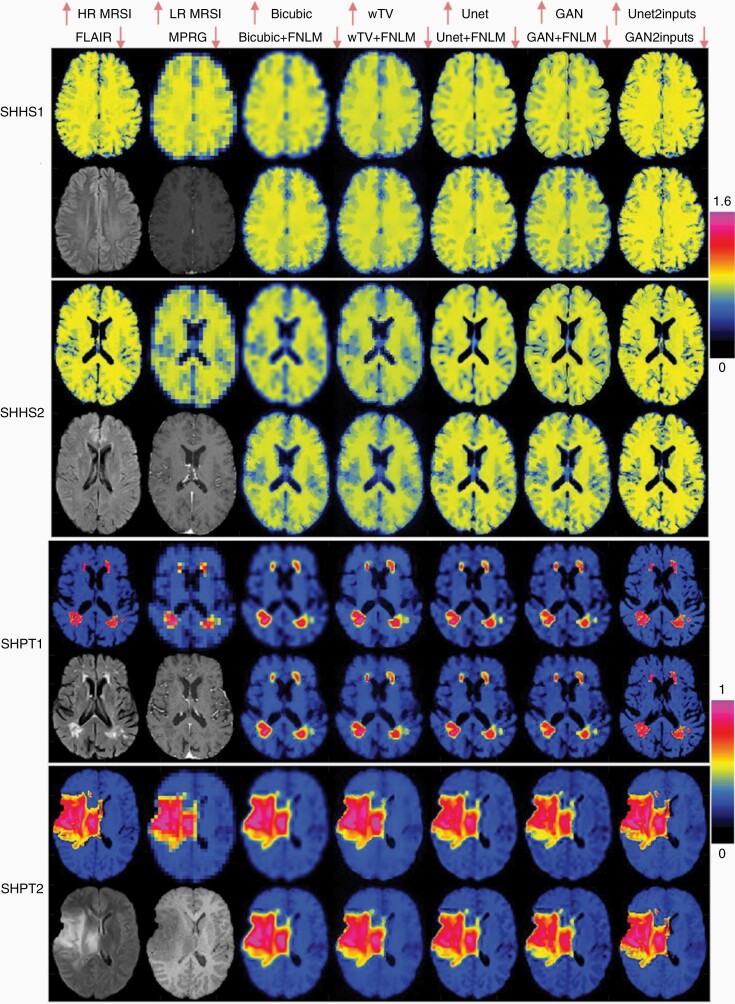
Super-resolution magnetic resonance spectroscopic imaging (MRSI) aided by prior MRI in simulated high-resolution NAA maps in healthy subjects (SHHS) data and simulated high-resolution d-2-hydroxyglutarate (2HG) maps in patients (SHPT) with glioma data. Results obtained by deep learning DLmethod_2 (Unet + FNLM↓, GAN + FNLM↓) and DLmethod_3 (↑Unet2inputs, GAN2inputs↓) are compared to DLmethod_1 (↑Unet, ↑GAN) and conventional (↑bicubic, ↑weighted TV w/wo FNLM↓) methods. Prior MRI is used to improve super-resolution MRSI, either by feature nonlocal means (FNLM) after neural networks, or as a second input (2 inputs) in the neural networks. Examples from 2 simulated healthy subjects and 2 simulated patients are shown. High-resolution (HR) ground truth MRSI (1.3 × 1.3 mm^2^), low-resolution (LR) MRSI (5.2 × 5.2 mm^2^), upsampled MRSI (1.3 × 1.3 mm^2^), and anatomical MRI (FLAIR and MPRAGE [MPRG] at 1 × 1 mm^2^). Up and down arrows by the names of the top of the figure indicate images in the upper or lower row, respectively, for a given subject.

Upsampling results combining deep learning and prior MRI (DLmethod_2 and DLmethod_3) are shown in Figure 3. For comparison we selected to show the maps for the same healthy and patient subjects used in the case of deep learning alone (DLmethod_1). Zoomed images of the tumor regions are shown in Supplementary Figure 3. The use of prior anatomical information in the SR pipeline, either sequentially in the form of FNLM (Unet/GAN + FNLM) or input simultaneously with MRSI in the DNNs (Unet2inputs/GAN2inputs) results in more clear structural details of the brain anatomy compared to DLmethod_1. The use of FNLM combined with deep learning (DLmethod_2) provides very close results to the ground truth images with good delineation of internal gray and white matter anatomy. The results obtained by simultaneous input of MRSI and prior MRI into neural networks (DLmethod_3) show the most anatomical details for SR MRSI, which are even finer higher compared to ground truth HR MRSI. This is visible in particular at the sulci of SR MRSI, which resemble more the appearance of sulci in the prior MRI than in the ground truth HR MRSI. This may represent overfitting of sulci by DLmethod_3. The results obtained by deep learning without prior MRI are close to the results of conventional methods combined with prior MRI. Maps obtained by GAN provide sharper structural details than Unet, similar to results of DLmethod_1.

Quantitative analysis of the image qualitative metrics is summarized in Table 2 and Supplementary Figure 4. Note that, although the first 4 methods are identical in Tables 1 and 2, the numerical scores are different because the ground truth images were differently simulated as mentioned in the methods. PSNR, SSIM, and FSIM show that deep learning methods outperform conventional methods, either alone or in combination with prior MRI. Deep learning without prior MRI scored significantly better than conventional interpolation methods alone (bicubic, wTV). The MOS of GAN is slightly higher than Unet without prior MRI, and close to the conventional methods with prior MRI (bicubic + FNLM and wTV + FNLM). It is remarkable that deep learning without prior MRI can infer structural details that are obtained when conventional methods are aided by prior MRI. Finally, deep learning combined with prior MRI scores the highest, with slightly higher scores for simultaneous input of MRSI and prior MRI. Additionally, we present the training loss curves for Unet and GAN in Supplementary Figure 5 to show the convergence of the training. However, we do not generate validation data which are used for training, considering the fact that it is more common to observe the samples generated after the completion of the training instead of checking the trend of the validation losses for GAN. Indeed, the quality of the generated samples helps determine the generator’s ability to learn a diverse representation of the input data distribution.

**Table 2.** Performance of deep learning methods combined with prior MRI (DLmethod_2 and DLmethod_3) for upsampling MRSI

Method→	Conventional		DLmethod_1		Conventional + MRI		DLmethod_2		DLmethod_3	
IQM↓	Bicubic	wTV	UNet	GAN	Bicubic + FNLM	wTV + FNLM	UNet + FNLM	GAN + FNLM	UNet2 inputs	GAN2 inputs
PSNR	28.09	27.93	28.90	27.94	28.59	28.08	28.97	28.00	30.83*	30.75*
SSIM	0.880	0.883	0.899	0.874	0.907	0.904	0.915	0.905	0.906	0.902
FSIM	0.854	0.887	0.910	0.892	0.898	0.899	0.920	0.906	0.921*	0.917*
MOS	3.02	3.13	3.63	3.84	4.07	4.06	4.42	4.36	4.57*	4.58*

Image quality metrics (IQM) of super-resolution MRSI were calculated relative to the ground truth high-resolution MRSI. Note that ground truth used for the methods in Table 2 was simulated differently than ground truth for Table 1. The largest values are shown in bold. Values that are statistically significant for deep learning compared to the best conventional methods (Bicubic + FNLM and wTV + FNLM) are indicated by asterisk. Boxplots of IQM are shown in Supplementary Figure 4. FSIM, feature similarity index measure; MOS, mean opinion score; MRSI, magnetic resonance spectroscopic imaging; PSNR, peak signal-to-noise ratio; SSIM, structure similarity index measure.

**Figure 4.**
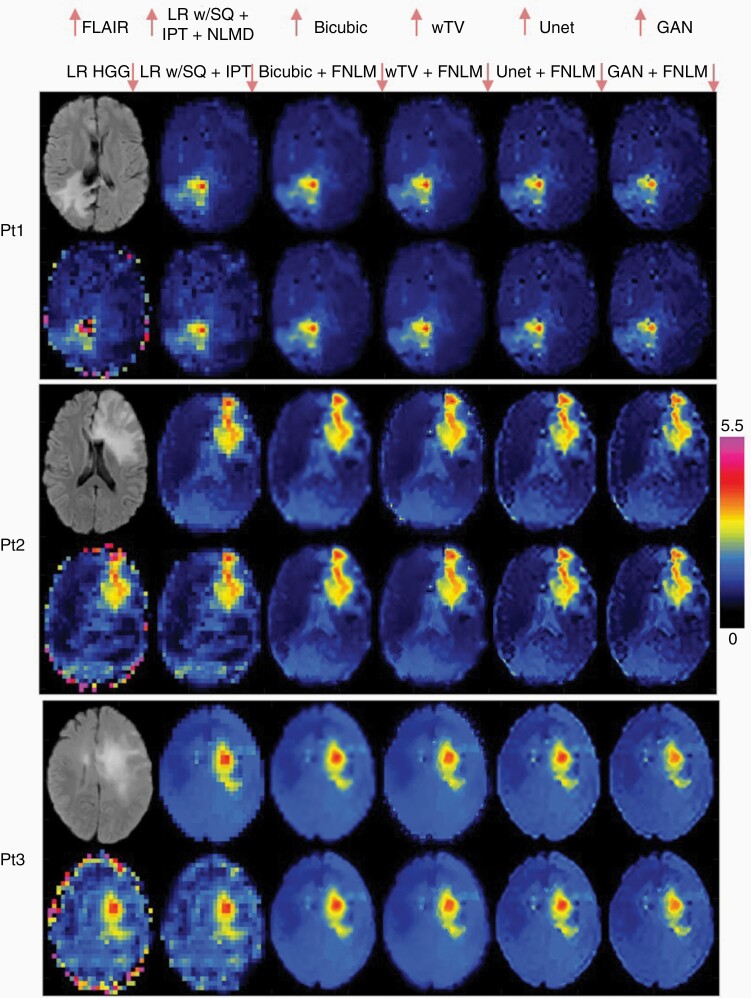
In vivo super-resolution magnetic resonance spectroscopic imaging (MRSI) measured in glioma patients (Pt). Original low-resolution HGG maps measured with the size 46 × 46 (5.2 × 5.2 mm^2^) were upsampled to 184 × 184 (1.3 × 1.3 mm^2^) with the corresponding methods from Figure 1. First, the low-resolution (LR) maps are filtered by spectral quality (SQ), inpainted for missing voxels (IPT), and denoised by nonlocal means denoising (NLMD). After denoising, MRSI is upsampled either by bicubic interpolation, weighted total variation, UNet, or GAN. Anatomical FLAIR images are used as prior to obtain super-resolution MRSI by feature nonlocal means (FNLM). Up and down arrows by the names of the top of the figure indicate images in the upper or lower row, respectively, for a given subject.

Applications of all the deep learning methods to in vivo measured data are presented in Figure 4 and Supplementary Figures 6 and 7. Metabolic maps obtained in 3 representative patients with mutant IDH glioma are shown in Figure 4 and Supplementary Figure 6, respectively. Sharper tumor boundaries are obtained by the Unet and GAN compared to bicubic and wTV. The heterogeneity of metabolic alterations inside the tumor are more evident by Unet and GAN. Results from 3 representative healthy volunteers in Supplementary Figure 7 show similar gradual improvement of structural details going from conventional bicubic interpolation to DNNs with prior MRI. Structural boundaries and edges appear sharper in GAN compared to Unet. Deep learning alone without prior MRI provides structural details similar to those obtained by combining conventional interpolation and prior MRI. Including prior MRI by FNLM to GAN and Unet improves anatomical details, but less than the improvement from conventional interpolation to deep learning without prior MRI. Simultaneous input of MRSI and prior MRI in DNNs results in images with most structural detail, in particular the sulci are emphasized in all subjects, and in subject 1 the boundary of gray–white matter becomes more apparent.

## Conclusions and Discussion

Our results indicate that the proposed deep learning methods can effectively enhance the spatial resolution and structural details of metabolite maps. This can be further improved by the aid of HR anatomical MR images. Deep learning methods without prior MRI (DLmethod_1) recover structural information and tissue contrast better than conventional interpolation methods (bicubic or wTV). The performance of DNNs without prior MRI, in particular GAN, is close to combination of conventional methods and FNLM prior MRI. This is particularly useful for patient data where lesions may appear differently in metabolic images versus anatomical images. The input of prior MRI together with MRSI in DNNs (DLmethod_3) provides the highest structural detail in healthy volunteers, however in patients features of the anatomical lesions may be inadvertently fused to the metabolic lesions when the 2 lesions are truly dissimilar at high resolution. In this regard, use of deep learning methods with MRSI data alone may avoid this unwanted effect in patients, while providing important structural improvements. Deep learning methods alone may be useful also in the case there is motion between the MRSI and MRI. The use of MRI to upsample MRSI assumes that the 2 data are well aligned, which may not be true if the subjects have moved between the 2 scans. Due to limited spatial resolution of MRSI it is hard to correct for motion in postprocessing.^35^ Alternatively, further improvements of deep learning metabolic maps (DLmethod_1) can be obtained by using FNLM and prior MRI (DLmethod_2). This hybrid approach is less susceptible to introduce spurious features and could be a safer choice in using prior anatomical MRI to increase spatial resolution of MRSI in glioma patients. DLmethod_3 may be more appropriate in healthy subjects and patients with diffuse brain involvement where metabolites and anatomy follow a more similar spatial pattern.

There is a small discrepancy between MOS and other image quality metrics in evaluating Unet and GAN results. Unet tends to have slightly higher PSNR, SSIM, and FSIM, while GAN has slightly higher MOS scores. This is not completely unexpected especially for subtle image differences such as between Unet and GAN. Image quality metrics have limitations, and it is hard to design a comprehensive metric that encompass all the perception details important for the human visual system.^34^ In particular, the human experts found that GAN provided sharper boundaries for brain structure and tumors, while with Unet the boundaries between different structures are slightly smoother.

The results for the MRSI data acquired in glioma patients suggest that the proposed methods have great clinical potential for guiding neurosurgery, radiation, and chemotherapy to deliver precision oncology healthcare. Determining tumor margins is important for the planning of surgery and radiation, in particular for mutant IDH gliomas where maximal resection and radiotherapy benefits patient overall survival.^36,37^ Objective treatment response to standard chemotherapy^8^ or targeted mutant IDH inhibitors^10^ can be assessed comprehensively by imaging tumor metabolism with sufficient structural detail to probe regional changes and tumor volume. Metabolic imaging brings more specificity to mapping of the tumors, which could disambiguate confounding effects of response and progression in anatomical imaging.

Our preliminary results in patients are promising, but further validation and verification of the proposed methodology are necessary. The main limitation in validation is the lack of sufficient MRSI data acquired at high resolution. Recently, very advanced acquisition methods have been developed,^26–29^ however their use is limited at the moment by very complex pulse sequences and reconstruction techniques which are available to only a small group of investigators. As these acquisition methods continue to disseminate more data will become available for training and validation. On the other hand, even if advanced sequences become more widely available the acquisition time of HR MRSI will still be long for many routine clinical exams and SR methods as shown here will be beneficial in reducing scan times in patients. Although, we demonstrated SR MRSI in glioma patients, our framework is generally applicable to other neurological or psychiatric diseases where metabolic imaging is important to probe disease mechanisms.

## Supplementary Material

vdac071_suppl_Supplementary_Material
