# Supplementary material for: Deep learning super-resolution magnetic resonance spectroscopic imaging of brain metabolism and mutant isocitrate dehydrogenase glioma
Source: Neurooncol Adv. 2022 May 24;4(1):vdac071. doi: 10.1093/noajnl/vdac071 (PMC9332900; doi:10.1093/noajnl/vdac071)
Supplement: vdac071_suppl_Supplementary_Material [file vdac071_suppl_supplementary_material.docx]

**Supplementary Material for “Deep Learning Super-resolution MR Spectroscopic Imaging of Brain Metabolism and Mutant IDH Glioma”**

1. **MRSI acquisition, reconstruction and processing.**

3D MRSI was acquired with TR/TE = 1800/97 ms, FOV = 240×240×120 mm^3^, matrix of 46×46×10, nominal voxel size of 5.2×5.2×12 mm^3^, 3 averages, acquisition time 18:22 min:s. Further details of pulse sequence and acquisition parameters are specified in (Li et al., 2020). In addition, anatomical MRI was performed with MPRAGE (FOV = 256×256×176 mm^3^, matrix = 256×256×176), FLAIR (FOV = 220×186×138 mm^3^, matrix = 256×192×23) and B0 fieldmap with a double gradient echo sequence (FOV = 240×240×120 mm^3^, matrix = 138×138×40).

MRSI k-space data were reconstructed with the following steps: 1) coil combination using a sensitivity map for spatial normalization ^1^; 2) phase correction for time evolution along spiral k-space trajectories points ^2^ ; 3) density compensation for the non-uniform k-space weighting of spirals ^3^; 4) gridding to Cartesian k-space using a Kaiser-Bessel kernel ^4^; 5) Hamming filtered in k-space; 6) residual lipid removal with L1 norm regularization ^5^; 7) Fourier transformation; 8) overdiscrete B0 field correction ^6^; 9) frequency alignment; 10) phase correction; 11) baseline correction; 12) and spectral fitting. The last four steps were performed as part of LCModel ^7^ spectral fitting, which used a simulated basis set that included D-2-hydroxyglutarate and twenty normal brain metabolites (Alanine, Ascorbate, Aspartate, Creatine, γ-Aminobutyric acid, Glycerophosphocholine, Glutathione, Glucose, Glutamine, Glutamate, Glycine, myo-Inositol, Lactate, N-acetylaspartylglutamate, N-acetyl-aspartate, Phosphocholine, Phosphocreatine, scyllo-Inositol, Serine, Taurine). Values from all voxels fitted by LCModel were used to obtain brain maps for metabolite levels, Cramer-Rao lower bounds (CRLB) goodness of fit, linewidth (LW) and signal-to-noise ratio (SNR). Ratios of metabolic maps were calculated in patients. In particular, two metabolic ratios have been shown (Li et al., 2020) to have high contrast-to-noise (CNR) for glioma tumors: 1) the HGG ratio between 2HG, glutamine and glutamate (HGG = ([2HG]+[Gln])/[Glu]), and 2) the TCN ratio between total choline and total NAA (TCN = [tCh]/[tNAA]).

Metabolic maps were corrected for voxels with unreliable metabolite fitting and denoised. Voxels that do not fulfill spectral quality criteria (CRLB <20%, LW <0.15 ppm, SNR >3) or considered outliers of the concentration confidence interval ([Metab] > mean + 5×stdev) were rejected from the low-resolution maps. In order to recover missing voxels we used an advanced image inpainting (IPT) algorithm that leverages both local and global information via discrete cosine transform and penalized least squares regression ^8,9^. Metabolic maps often show spurious signal variability between neighboring voxels due to the intrinsic low signal-to-noise of MRSI. To correct this effect we used a non-local means denoising (NLMD) method as described in ^10^. More details of the processing pipeline are given in ^10^.

**2. Feature based non-local means method.**

To further improve the SR results obtained by DLmethod_1, a feature non-local means method (FNLM) can be employed by using the available anatomical image prior information. In this DLmethod_2 approach the output of DLmethod_1 trained only on MRSI data becomes the input to the FNLM pipeline (DLmethod_2 = DLmethod_1 + FNLM). The advantage of combining deep learning and FNLM is twofold: 1) the FNLM method has been shown to be robust with regard to overfitting and introducing false features in patient metabolic maps when the spatial support of tumors is different in MRSI vs MRI, and 2) the initialized image using deep learning methods can be a better starting point for FNLM than bicubic or total variation initialization.

FNLM combines patch-based interpolation with a feature vector representing the laminar pattern of the brain structure. FNLM is capable of finding similar voxels and estimates accurately the corresponding interpolating weights for metabolic maps as described in **^10^**. Based on the model [1], the feature non-local means can be formulated as the following optimization problem

$\underset{\boldsymbol{I}^{HR}}{\bar{\boldsymbol{I}^{HR}}= arg \min} R\left( \boldsymbol{I}^{HR} \right) subject to \left\| \boldsymbol{I}^{LR}-F^{-1}(\boldsymbol{I}^{HR}) \right\|^{2}\leq\varepsilon$ [4]

where $\varepsilon$ is a small positive number, and the regularization term $R(\cdot)$ is defined as

$R\left( \boldsymbol{I}^{HR} \right)=\sum_{\mathbf{v}} \left| \boldsymbol{I}^{HR}\left( \mathbf{v} \right)\mathbf{-}\sum_{\mathbf{u}\boldsymbol{\in}\mathbf{Ω}\boldsymbol{(}\mathbf{v)}} \mathbf{w}\boldsymbol{(}\mathbf{v}\boldsymbol{,}\mathbf{u}\boldsymbol{)}\boldsymbol{I}^{HR}\left( \mathbf{u} \right) \right|^{2}$ [5]

where $\mathbf{Ω}\boldsymbol{(}\mathbf{v)}$ is the patch neighborhood of voxel $\mathbf{v}$**,** and the voxel weights are calculated as

$\mathbf{w}\left( \mathbf{v},\mathbf{u} \right)\propto\mathbf{exp}^{\left( -(\alpha\sum_{i} \left\| \mathbf{F}_{\mathbf{i}}^{\mathbf{MRSI}}\left( \mathbf{v} \right)\mathbf{-}\mathbf{F}_{\mathbf{i}}^{\mathbf{MRSI}}\left( \mathbf{u} \right) \right\|^{2}+(1-\alpha)\sum_{i} \left\| \mathbf{F}_{\mathbf{i}}^{\mathbf{MRI}}\left( \mathbf{v} \right)\mathbf{-}\mathbf{F}_{\mathbf{i}}^{\mathbf{MRI}}\left( \mathbf{u} \right) \right\|^{2}) \right)},\mathbf{i}=1,2,\ldots$ n [6]

based on the feature vector components:$\mathbf{F}_{\mathbf{1}}^{\mathbf{MRI}}\left( \mathbf{v} \right)\boldsymbol{=}\boldsymbol{I}_{\mathrm{MRI}}^{\boldsymbol{HR}}\left( \mathbf{v} \right)$**,** $\mathbf{F}_{\mathbf{2}}^{\mathbf{MRI}}\left( \mathbf{v} \right)\boldsymbol{=}\boldsymbol{\nabla}\boldsymbol{I}_{\mathrm{MRI}}^{\boldsymbol{HR}}\left( \mathbf{v} \right)$**,**

$\mathbf{F}_{\mathbf{3}}^{\mathbf{MRI}}\left( \mathbf{v} \right)\boldsymbol{=} {\boldsymbol{I}_{\mathrm{MRI}}^{\boldsymbol{HR}}\boldsymbol{*}\mathbf{g}_{\boldsymbol{1}}\mathbf{|}}_{\mathbf{v}}$**,** $\mathbf{F}_{\mathbf{4}}^{\mathbf{MRI}}\left( \mathbf{v} \right)\boldsymbol{=}{\boldsymbol{I}_{\mathrm{MRI}}^{\boldsymbol{HR}}\mathbf{*}\mathbf{g}_{\mathbf{2}}\mathbf{|}}_{\mathbf{v}}$**,** $\mathbf{F}_{\mathbf{i}}^{\mathbf{MRI}}\left( \mathbf{v} \right)\boldsymbol{=}\boldsymbol{I}_{\mathrm{MRI}}^{\boldsymbol{HR}}\mathbf{*}\mathbf{g}_{\mathbf{j-2}}\boldsymbol{|}_{\mathbf{v}}$**, … ,** $\mathbf{j}=3,\ldots,n-2$,

where $\mathbf{*}$ is a convolutional operator,$\mathbf{g}_{\mathbf{j-2}}$**,** $\mathbf{j}=3,\ldots n-2$, are Gaussian kernels of different standard deviation.

**3.** **Supplementary Figures.**


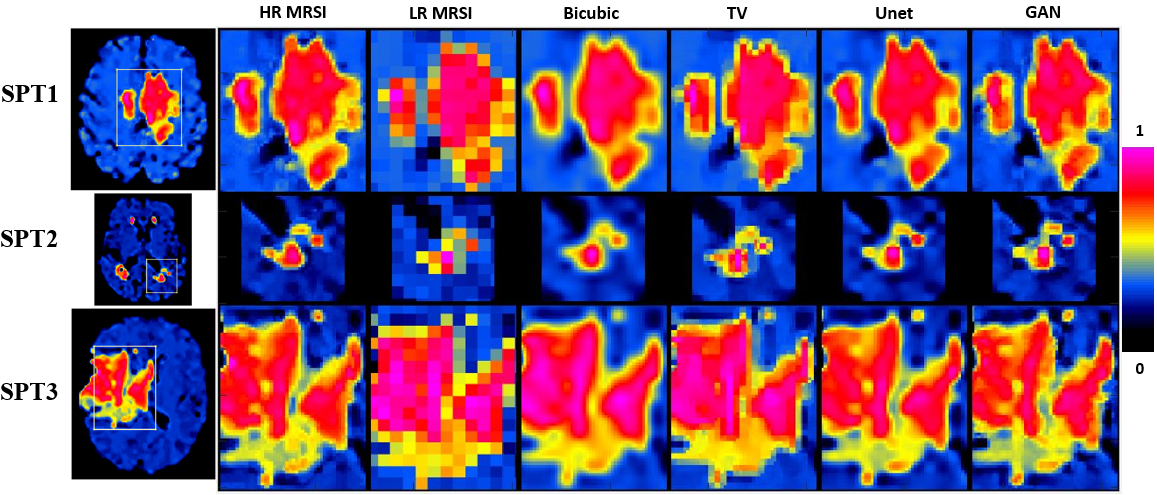


**Supplementary Figure 1.** Super-resolution MRSI without prior MRI (DLmethod_1). Zoom on the tumor region for the same patients shown in Fig 2. It is apparent that GAN provides sharper tumor margins than Unet. Both networks are superior to bicubic and TV.


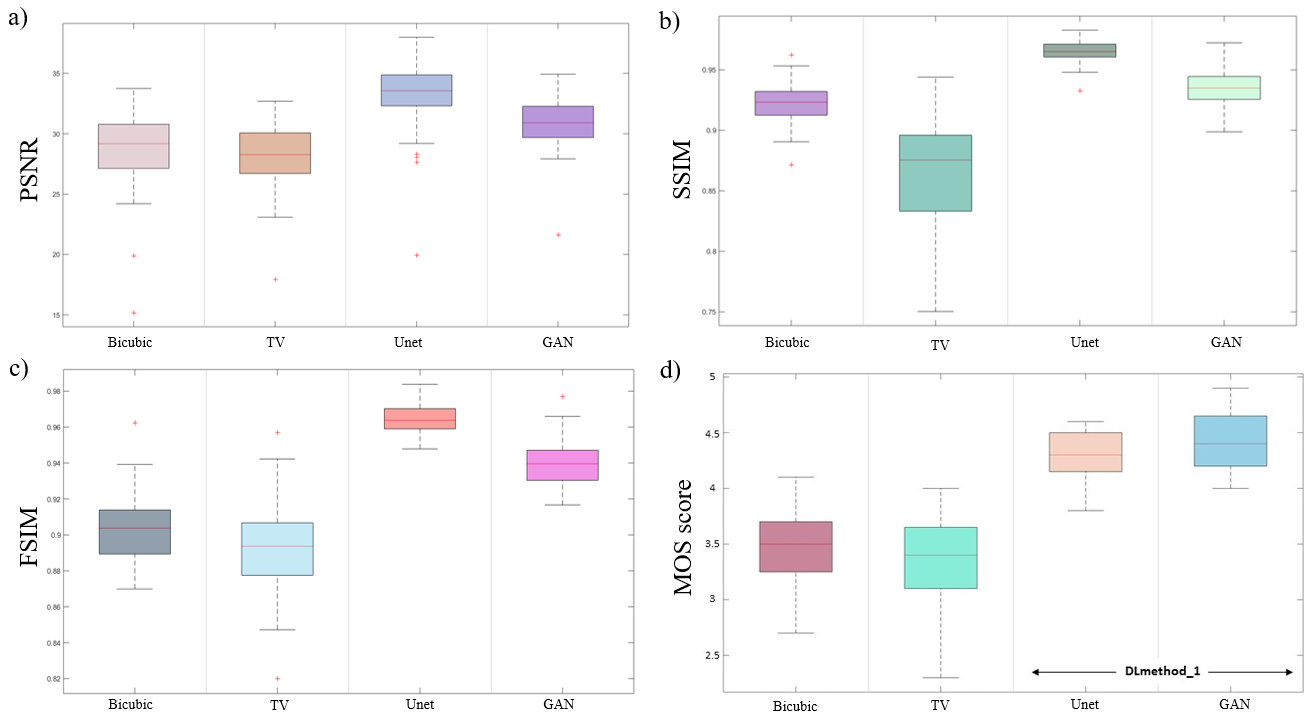


**Supplementary Figure 2**: Box plot of the peak signal-to-noise ratio (PSNR), structural similarity Index (SSIM), Feature similarity Index (FSIM), and the mean opinion scores (MOS) in all subjects for super-resolution MRSI without the use of prior MRI (DLmethod_1). Both Unet and GAN score significantly higher than conventional interpolation.


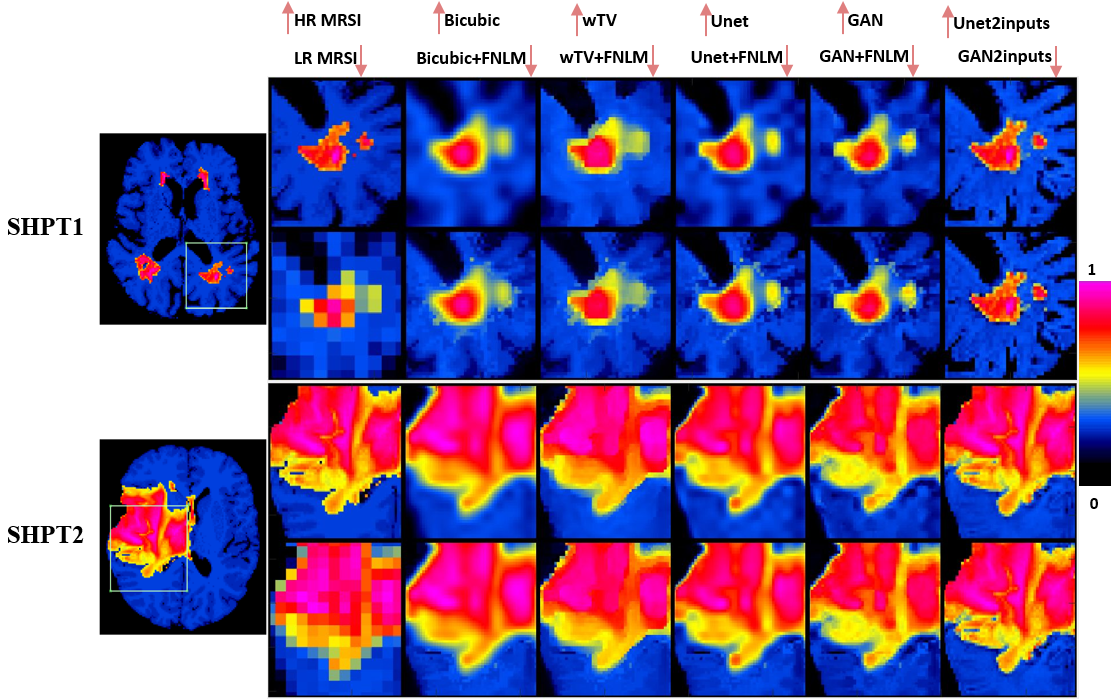


**Supplementary Figure 3.** Super-resolution MRSI aided by prior MRI (DLmethod_2 and DLmethod_3). Zoom on the tumor region for the same patients shown in Figure 3. DLmethod_2 (Unet+FNLM, GAN+FNLM) provides slight improvement over DLmethod_1 (Unet, GAN). The DLmethod_3 (Unet2inputs, GAN2inputs) provides the largest improvement and closest results to ground truth. Deep learning alone (DLmethod_1) is superior to conventional methods aided by prior MRI (Bicubic+FNLM, wTV+FNLM).


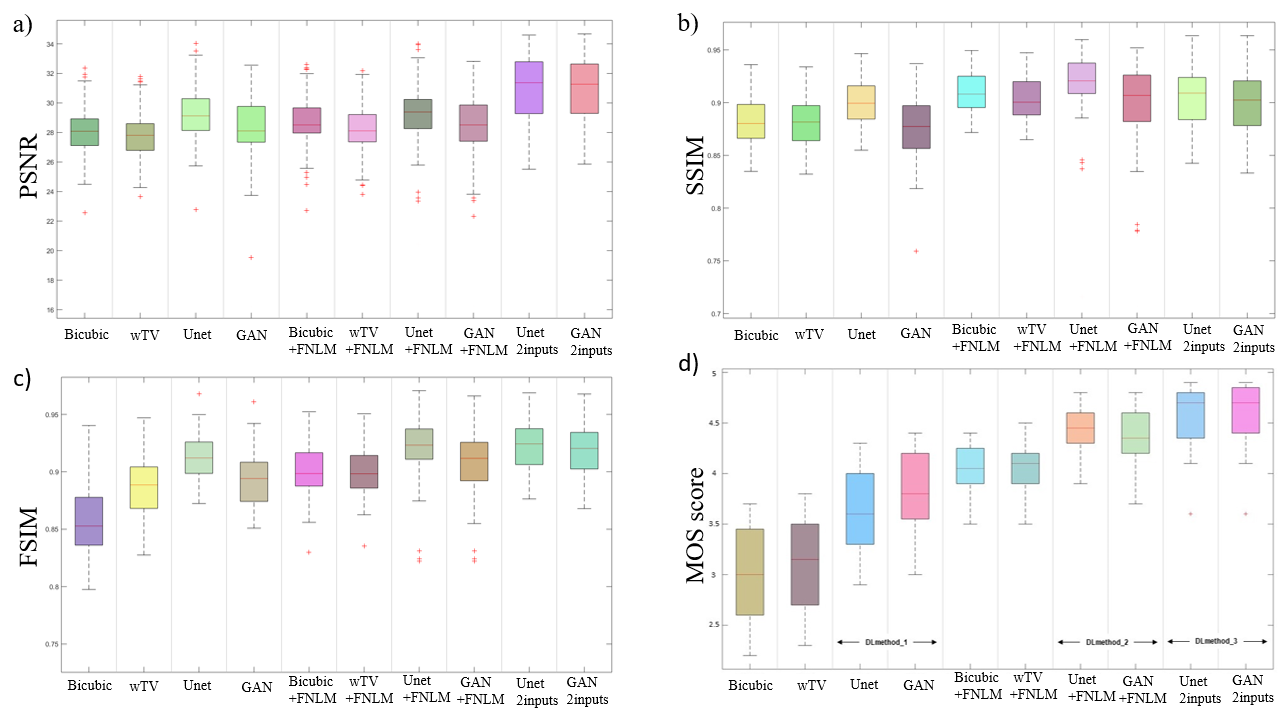


**Supplementary Figure 4**. Box plot of the peak signal-to-noise ratio (PSNR), structural similarity Index (SSIM), Feature similarity Index (FSIM), and the mean opinion scores (MOS) in all subjects for all super-resolution methods.


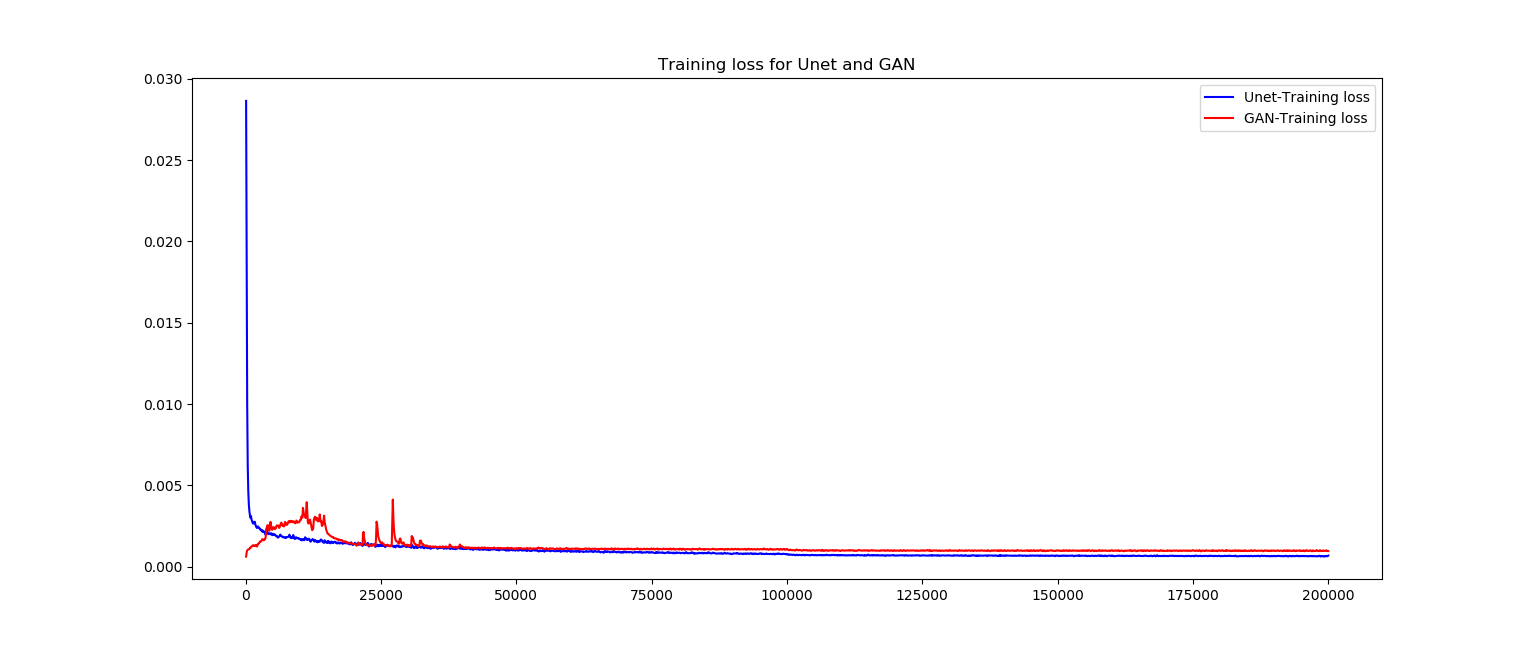


**Supplementary Figure 5**. The training loss curves for Unet and the generator of GAN after completion of training (200k iterations). Unet was used as a pretrained model for training GAN. The loss curve of the generator shows the adversarial process between generator and discriminator.


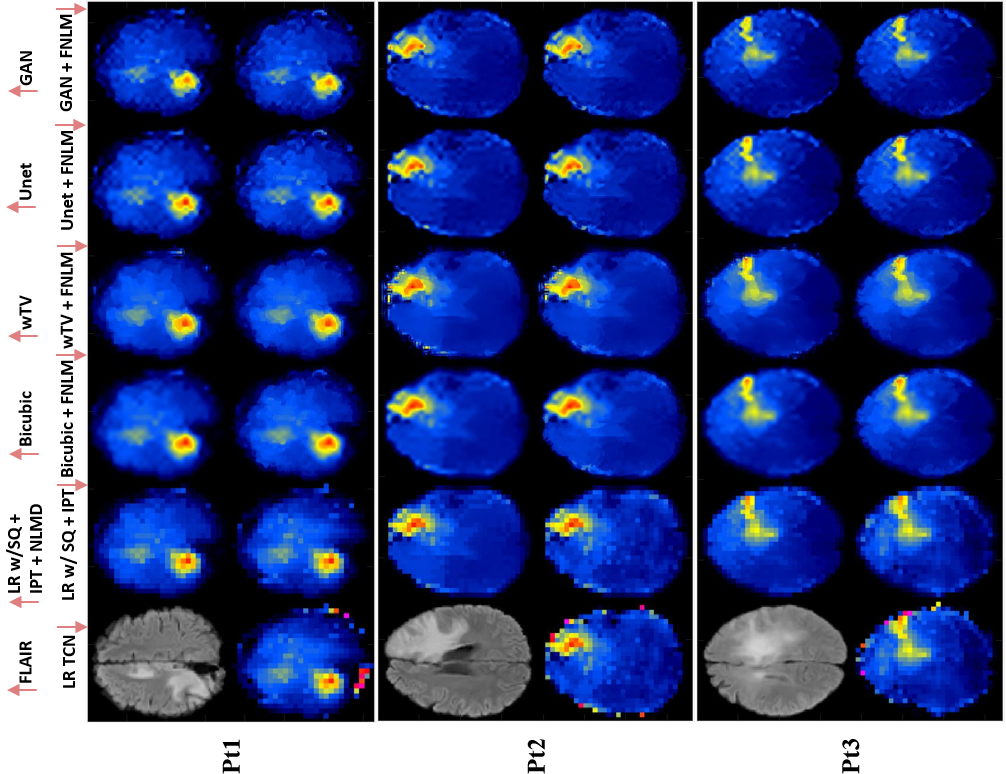


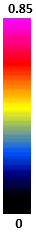


**Supplementary Figure 6**. *In vivo* super-resolution MRSI in glioma patients. Total choline to NAA (TCN) maps measured with the size 46$\times$46 (5.2$\times$5.2 mm^2^) were upsampled to 184$\times$184 (1.3$\times$1.3 mm^2^) with all the super-resolution methods. Before super-resolution the low-resolution (LR) maps are filtered by spectral quality (SQ), inpainted for missing voxels (IPT), and denoised by non-local means denoising (NLMD). Anatomical FLAIR images are used to improve super-resolution MRSI by feature non-local means (FNLM).


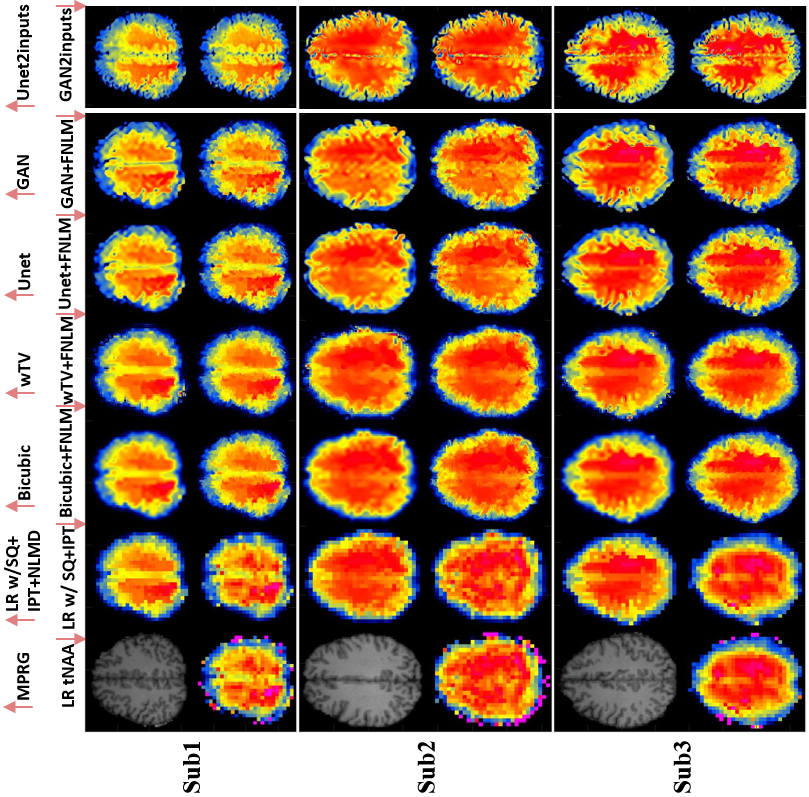


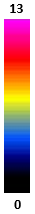


**Supplementary Figure 7**. *In vivo* super-resolution MRSI in healthy subjects. Total *N*-acetylaspartate (tNAA) maps measured with size 46$\times$46 (5.2$\times$5.2 mm^2^) were upsampled to 184$\times$184 (1.3$\times$1.3 mm^2^) with all the methods. Before super-resolution the low-resolution (LR) maps are filtered by spectral quality (SQ), inpainted for missing voxels (IPT), and denoised by non-local means denoising (NLMD). Anatomical MPRAGE (MPRG) images are used to improve super-resolution MRSI either by feature non-local means (FNLM) or input together in the deep neural networks (2inputs).

**References:**

**1.** Brown MA. Time-domain combination of MR spectroscopy data acquired using phased-array coils. *Magnetic resonance in medicine.* 2004; 52(5):1207-1213.

**2.** Mayer D, Levin YS, Hurd RE, Glover GH, Spielman DM. Fast metabolic imaging of systems with sparse spectra: application for hyperpolarized 13C imaging. *Magnetic resonance in medicine.* 2006; 56(4):932-937.

**3.** Hoge RD, Kwan RK, Pike GB. Density compensation functions for spiral MRI. *Magnetic resonance in medicine.* 1997; 38(1):117-128.

**4.** Beatty PJ, Nishimura DG, Pauly JM. Rapid gridding reconstruction with a minimal oversampling ratio. *IEEE transactions on medical imaging.* 2005; 24(6):799-808.

**5.** Bilgic B, Gagoski B, Kok T, Adalsteinsson E. Lipid suppression in CSI with spatial priors and highly undersampled peripheral k-space. *Magn Reson Med.* 2013; 69(6):1501-1511.

**6.** Kirchner T, Fillmer A, Henning A. Mechanisms of SNR and line shape improvement by B0 correction in overdiscrete MRSI reconstruction. *Magnetic resonance in medicine.* 2017; 77(1):44-56.

**7.** Provencher SW. Estimation of Metabolite Concentrations from Localized in-Vivo Proton Nmr-Spectra. *Magnetic resonance in medicine.* 1993; 30(6):672-679.

**8.** Garcia D. Robust smoothing of gridded data in one and higher dimensions with missing values. *Computational Statistics and Data Analysis.* 2010.

**9.** Torrado-Carvajal A, Albrecht DS, Lee J, et al. Inpainting as a Technique for Estimation of Missing Voxels in Brain Imaging. *Annals of biomedical engineering.* 2021; 49(1):345-353.

**10.** Li X, Strasser B, Jafari-Khouzani K, et al. Super-Resolution Whole-Brain 3D MR Spectroscopic Imaging for Mapping D-2-Hydroxyglutarate and Tumor Metabolism in Isocitrate Dehydrogenase 1-mutated Human Gliomas. *Radiology.* 2020; 294(3):589-597.
